# Case Report: Intracranial germ cell tumors in girls presenting with peripheral precocious puberty and arginine vasopressin deficiency

**DOI:** 10.3389/fendo.2026.1886797

**Published:** 2026-10-16

**Authors:** Wen Xin, Huamei Ma, Yanhong Li, Jun Zhang, Song Guo, Rujiang Zheng, Bing Wang, Huangmeng Xiao, Qiuli Chen

**Affiliations:** Department of Pediatrics, The First Affiliated Hospital, Sun Yat-Sen University, Guangzhou, China

**Keywords:** arginine vasopressin deficiency, diabetes insipidus, female, peripheral precocious puberty, β-HCG-secreting intracranial germ cell tumors

## Abstract

**Objective:**

This report summarizes the clinical characteristics of three female children with clinically diagnosed β-human chorionic gonadotropin (β-hCG)-secreting intracranial germ cell tumors (iGCTs) who presented with peripheral precocious puberty (PPP) and arginine vasopressin deficiency (AVP-D), with the aim of facilitating earlier diagnosis and treatment.

**Methods:**

Clinical data of the 3 female patients with iGCTs with initial symptoms of PPP and AVP-D were retrospectively analyzed, and the relevant literature was reviewed.

**Results:**

All the 3 female children first had polydipsia and polyuria, but their parents paid no attention or sought no medical help until the girls developed thelarche. The children were diagnosed with PPP and AVP-D. Moreover, their β-hCG levels were elevated in the peripheral blood (PB) and cerebrospinal fluid (CSF), and pituitary magnetic resonance imaging (MRI) revealed space-occupying lesions in the sellar region. Initial serum β-hCG concentrations in the 3 children were 5010.0, 314.3, and 202.8 mIU/mL, respectively. After regular chemotherapy and radiotherapy, β-hCG levels in the PB and CSF returned to normal, MRI scans revealed tumor reduction, Tanner stages regressed to stage I, and estradiol and testosterone levels decreased. Seven months after the treatment was completed, Patient 1 developed rapidly progressive central precocious puberty.

**Conclusion:**

In children of either sex presenting with precocious puberty accompanied by multiple pituitary dysfunctions, including AVP-D, or mass effect manifestations, such as decreased visual acuity, routine serum β-hCG and AFP testing and contrast-enhanced sellar MRI are recommended to exclude organic lesions, including iGCTs.

## Introduction

Peripheral precocious puberty (PPP) is less common than central precocious puberty (CPP) in children. It is caused by excessive secretion of sex hormones from tumors or by exogenous factors. It can also occur secondary to certain genetic diseases. PPP does not depend on the activation of the hypothalamic–pituitary–gonadal axis (HPGA), and the levels of gonadotropin-releasing hormone (GnRH), follicle-stimulating hormone (FSH), and luteinizing hormone (LH) are suppressed in patients with PPP (1, 2). Intracranial germ cell tumors (iGCTs) in children are among the causes of PPP. Primary iGCTs can be pathologically divided into germinomas and nongerminomatous germ cell tumors (NGGCTs). β-Human chorionic gonadotropin (β-hCG) is an important marker for iGCTs and can partially indicate the pathological type of the tumor. For example, choriocarcinoma, embryonal carcinoma, and immature teratoma secrete large amounts of β-hCG, whereas germinomas secrete small amounts of β-hCG. Nevertheless, pathological examination remains the gold standard for diagnosis. β-hCG-secreting iGCTs are a recognized cause of PPP, although this presentation is much more common in boys than in girls (3–5). Due to the low follicle-stimulating hormone (FSH)-like activity of β-hCG, stimulation of ovarian function is limited, which may partly account for the rarity of PPP in girls; therefore, female children with iGCTs rarely present with PPP (6). In addition to PPP, in some rare cases, iGCTs may lead to CPP in both boys and girls through space-occupying effects (6). Although individual cases of PPP in female children have been reported worldwide, the association between iGCTs and precocious puberty warrants further investigation.

This article summarizes the clinical characteristics of 3 female children with clinically diagnosed β-hCG-secreting iGCTs who presented with PPP and arginine vasopressin deficiency (AVP-D) and provides a comprehensive review and integration of the available literature.

## Patients and methods

### Patients

This retrospective study evaluated the clinical characteristics of 3 female children with iGCTs presenting with PPP and AVP-D who were admitted to the Department of Pediatric Endocrinology at The First Affiliated Hospital, Sun Yat-sen University, between 2020 and 2025.

### Methods

Clinical data of the patients were extracted from electronic and paper medical records. The relevant patient information included demographic data (sex and age), initial symptoms, physical examination results, laboratory test results, treatment, and follow-up data.

### Literature search

A literature search was conducted in the PubMed, Wanfang, and China National Knowledge Infrastructure (CNKI) databases by using the terms “intracranial germ cell tumors”, “precocious puberty”, “arginine vasopressin deficiency”, “diabetes insipidus”, and “β-HCG” as keywords. The clinical characteristics of female children with iGCTs who presented with PPP and AVP-D (known as diabetes insipidus), as reported in the literature, were collected and summarized.

## Results

### Patient presentation

All the 3 girls developed the disease at approximately 5–6 years of age. At onset, they first displayed polydipsia and polyuria for 1 year, 5 months, and 1 year and 5 months, respectively; these symptoms were not given sufficient attention. They subsequently developed thelarche and sought medical help. None of the children had central nervous system symptoms such as headache, blurred vision or vomiting. The physical examination and laboratory test results of the 3 girls at admission are shown in **Table 1**.

**Table 1:** Physical examination and laboratory test results at admission.

Items	Case 1	Case 2	Case 3	Normal Range
Age at onset (years)	5.75	6.08	5	-
Age at diagnosis (CA, years)	6.75	6.5	6.25	-
Bone age at diagnosis (BA, years)	6	4.83	4.17	-
Height (cm)	112	111.7	105.7	-
Height for age (SD)	-1.9	-1.5	-2.7	−2~+2SD
Weight (kg)	17.2	16.8	13.9	-
Weight for age (SD)	-2	-1.9	<-3	−2~+2SD
Tanner stage	A2, B3, PH2	A1, left B3, right B2, PH1	A1, B2, PH1	Prepubertal girl: A1, B1, PH1
Urine volume (mL/m^2^)	4076.1	4189.9	4651.2	≤2000 ml/m²/24 h (27)
Serum osmolality (mOsm/L)	282	306	283	275~295 (28)
Urine osmolality (mOsm/kg)	63	106	113	>200 (29)
Serum Sodium (mmol/L)	138	149	138	135-145
T (nmol/L)	< 0.45	0.52	0.49	0.04~2.15 (30)
E2 (pmol/L)	99.1	<36.7	55.1	0-36.7 (30)
PRL (nmol/L)	0.16	4.14	1.89	0.18~1.00 (30)
LH (IU/L)	0.04	0.04	0	0-0.33 (30)
FSH (IU/L)	< 0.05	<0.05	<0.05	0.42-5.45 (30)
8 AM ACTH (pmol/L)	3.53	2.53	2.93	1.6–13.9^a^
8 AM Cortisol (nmol/L)	416.6	96.6	104.8	49.6–323.7 (31)
Androstenedione (nmol/L)	4.10	<1.05	2.11	0.35−1.43 (32)
Dehydroepiandrosterone (μmol/L)	1.21	0.32	ND	0.02–1.11 (33)
17α-Hydroxyprogesterone (nmol/L)	7.62	0.51	ND	0.30−4.84 (32)
TSH (mIU/L)	1.90	0.915	2.539	0.83–7.42 (34)
FT3 (pmol/L)	-	2.78	3.62	3.77−6.80 (34)
FT4 (pmol/L)	11.21	8.352	7.17	13.26–24.80 (34)
IGF-1 (ng/mL)	321.00	75.3	<15.0	35−232^a^
PB β-hCG (mIU/mL)	5010.0	314.30	202.8	<1.0
CSF β-hCG (mIU/mL)	2120.7	1147.60	283.3	Negative
AFP(ug/L)	2.4	2.2	3.4	0-20
Pituitary MRI	Suprasellar germ cell tumor (23 mm×20 mm×24 mm)	Uniform thickening of the pituitary stalk (approximately 5 mm in width)	Hypothalamic germ cell tumor (30 mm×27 mm×32 mm)	normal

ND, not done; a, our laboratory kit normal range.

### Diagnosis

In combination with clinical symptoms, physical examination, and auxiliary examinations, all three patients were clinically diagnosed with iGCTs, PPP, and AVP-D; none had histopathological confirmation. Cases 2 and 3 were complicated with hyperprolactinemia, central hypothyroidism, and central adrenal insufficiency. Case 2 demonstrated pituitary stalk thickening accompanied by AVP-D. Differential diagnoses included Langerhans cell histiocytosis, pilocytic astrocytoma, and craniopharyngioma. While these pathologies may affect the hypothalamic-pituitary axis and result in endocrine dysfunction, substantial elevations in both serum and cerebrospinal fluid (CSF) β-hCG are atypical for these entities. Accordingly, even in the absence of histopathological verification, the constellation of a pituitary-stalk lesion and markedly raised β-hCG concentrations in serum and CSF strongly favored a clinical diagnosis of β-hCG-secreting intracranial germ-cell tumor. Moreover, case 3 presented with growth retardation (height-for-age SDS: −2.7) and underweight (weight-for-age SDS: <−3). Case 3 exhibited markedly decreased IGF-1 levels. Although low IGF-1 concentrations may result from malnutrition, chronic disorders and other conditions, and a growth hormone (GH) stimulation test was not performed in this patient, she presented with severe growth retardation, confirmed structural lesions in the hypothalamic-pituitary region, and multiple additional pituitary hormone insufficiencies. These findings substantially increase the likelihood of growth hormone deficiency (GHD). Current guidelines recommend that GH provocative testing may be omitted to establish a GHD diagnosis in short-stature patients with organic hypothalamic–pituitary lesions combined with multiple pituitary hormone deficiencies (7). Accordingly, GHD was diagnosed in Case 3.

### Treatment and outcomes

After admission, all the 3 patients received chemotherapy and radiotherapy. Changes in their clinical conditions during treatment are summarized in **Table 2**.

**Table 2:** Treatment and clinical changes in the 3 patients.

Items	Case 1	Case 2	Case 3
Chemotherapy regimen	4 cycles of the EP regimen + 2 cycles of the IE regimen	3 cycles of the EP regimen + 3 cycles of the IE regimen	4 cycles of the CP regimen + 2 cycles of the IE regimen
Pituitary hormone replacement therapy	Hydrocortisone, fludrocortisone and desmopressin	Hydrocortisone, desmopressin and levothyroxine sodium	Hydrocortisone, desmopressin and levothyroxine sodium
Complications during treatment	Cerebral salt-wasting syndrome	Ifosfamide encephalopathy	-
Time to regression of breast and pubic hair to Tanner Stage I	After 2 chemotherapy cycles	After 2 chemotherapy cycles	After 2 chemotherapy cycles
Time to normalization of PB and CSF β-hCG levels	After 3 chemotherapy cycles	After 2 chemotherapy cycles	After 2 chemotherapy cycles
Sex hormone decline	Yes	Yes	Yes
Tumor shrinkage	Yes	Yes	Yes
Radiotherapy after chemotherapy	Yes	Yes	Yes

a. EP regimen: cisplatin + etoposide; b. IE regimen: ifosfamide + etoposide; c. CP regimen: carboplatin + etoposide. In case 1, cerebral salt wasting occurred after the second ifosfamide-free EP cycle and before initiation of the IE regimen. Recurrent hyponatremia (Na 122 mmol/L), increased urinary sodium excretion (476.15 mmol/24 h; fractional excretion of sodium: 33%), increased urine output and hypouricemia (62 μmol/L), together with improvement after fludrocortisone treatment, supported the diagnosis of cerebral salt wasting.

In case 1, hydrocortisone and fludrocortisone were voluntarily discontinued following radiotherapy completion on the basis of two consecutive normal biochemical test results from local re-evaluations, whereas desmopressin was continued. Subsequent biochemical follow-ups remained unremarkable. Seven months after the end of radiotherapy, when the child was 7 years and 9 months old, breast development was accompanied by accelerated growth (annualized growth rate: 13 cm/year). Physical examination revealed Tanner stages A2, B3, and PH2. The results of the GnRH stimulation test revealed an FSH concentration of 12.51 IU/L and an LH concentration of 15.04 IU/L at 60 min, with an LH/FSH ratio of 1.2. Re-examination revealed normal β-hCG levels in the CSF and PB, decreased sex hormone levels compared with those before, and no significant changes in cranial MRI results (**Figure 1**). Bone age progressed from 6.5 years to 7 years and 10 months within 3 months, with ΔBA/ΔCA = 5. Therefore, rapidly progressive CPP was diagnosed, and GnRH agonist treatment (2.78 mg·q4w) was administered. In addition, in cases 2 and 3, regular post-treatment re-examinations showed normal β-hCG levels in the CSF and PB, while cranial MRI demonstrated tumor disappearance. Nevertheless, both patients continued to require hormone replacement therapy (**Figures 2**, **3**).

**Figure 1:**
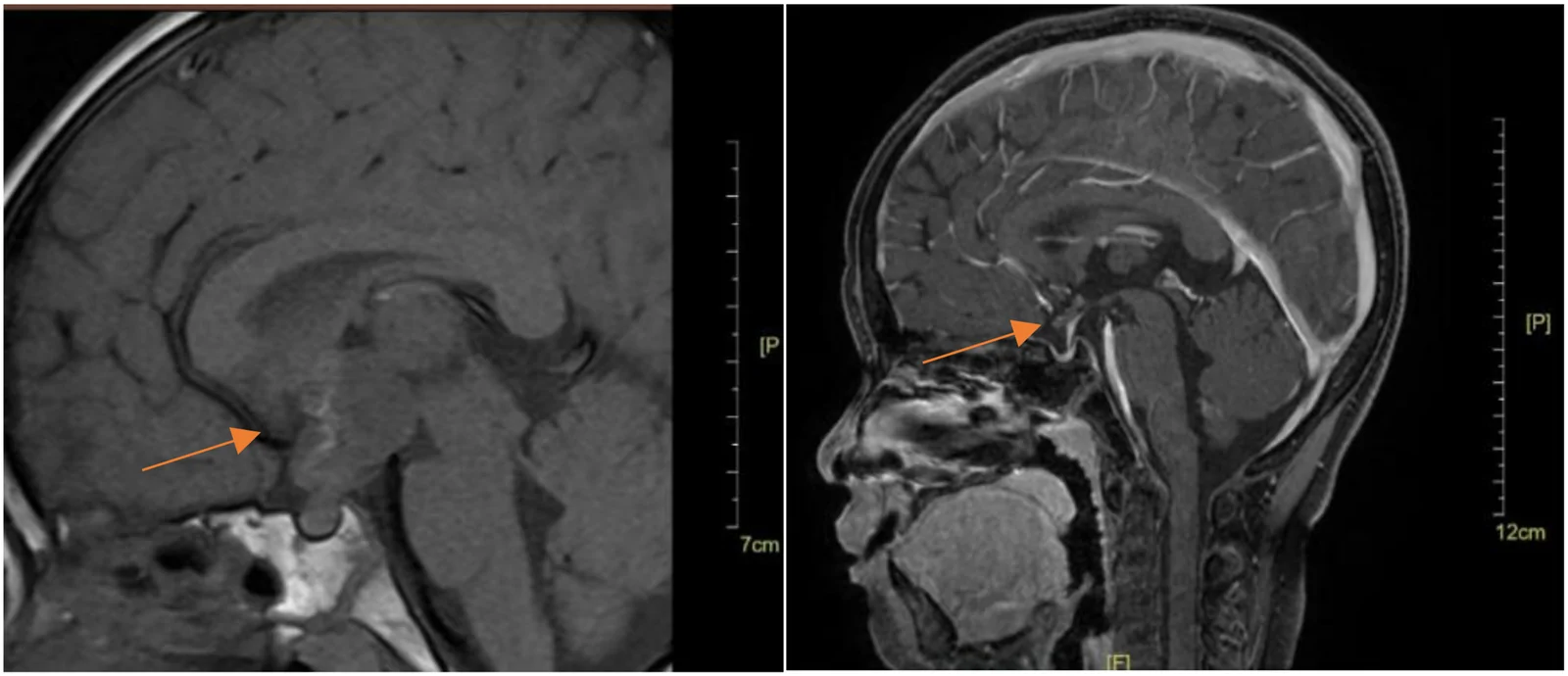
Cranial MR images of case 1 before treatment (left, suprasellar germ cell tumor 23 mm×20 mm×24 mm with cystic degeneration, necrosis and hemorrhage, involving the optic chiasm and pituitary stalk, extending into the sella turcica along the pituitary stalk, and involving the fornix and left basal ganglia region) and after treatment (right).

**Figure 2:**
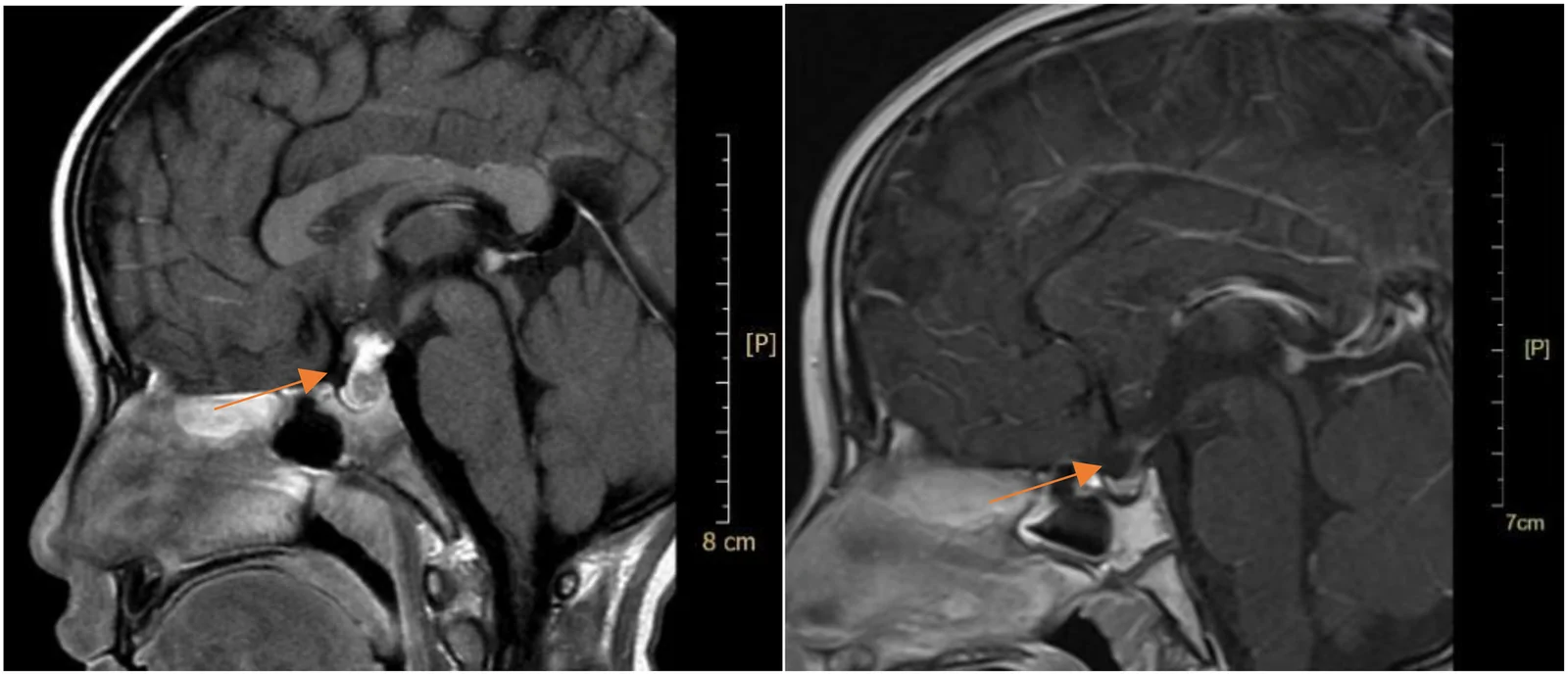
Cranial MR images of case 2 before treatment (left, uniform thickening of the pituitary stalk 5 mm in width with cystic degeneration in the lower segment of the pituitary stalk 5.6 mm×5.8 mm×8.5 mm) and after treatment (right).

**Figure 3:**
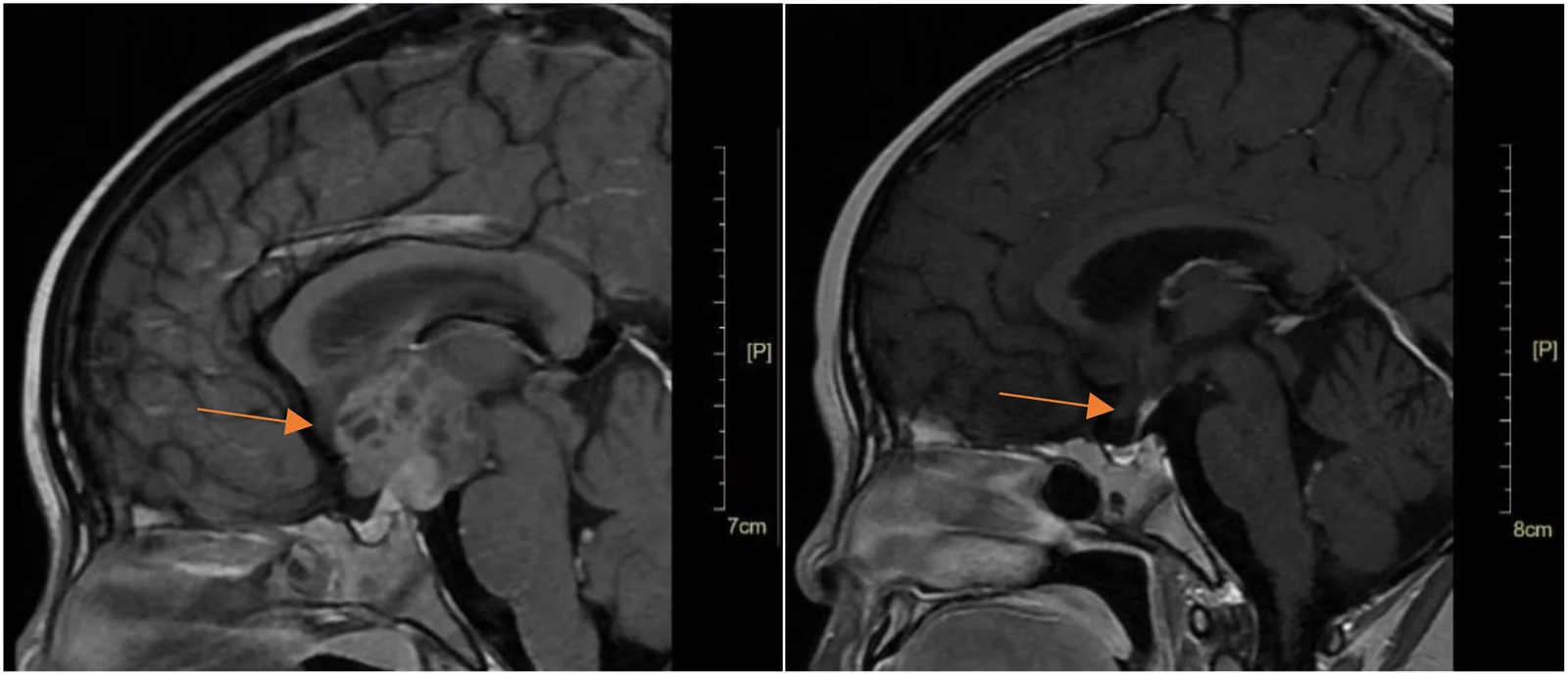
Cranial MR images of case 3 before treatment (left, hypothalamic germ cell tumor 30 mm×27 mm×32 mm involving the pituitary stalk, posterior pituitary lobe, optic chiasm and intracranial segments of the bilateral optic nerves, compressing the third ventricle, with slight symmetrical widening of the bilateral lateral ventricles) and after treatment (right).

## Literature review and discussion

Primary iGCTs are common intracranial tumors in children, accounting for approximately 3–15% of primary pediatric intracranial neoplasms (6). Their clinical manifestations include symptoms of increased intracranial pressure (such as headache, vomiting, and altered consciousness); symptoms caused by compression of adjacent brain structures (such as ocular movement disorders, tinnitus, hearing loss, ataxia, drowsiness, and obesity), particularly when the superior colliculus, tectum, inferior colliculus, medial geniculate body, or hypothalamus is involved; and endocrine disorders, such as precocious puberty and AVP-D (8). PPP associated with β-hCG-secreting iGCTs occurs predominately in male children and is rare in female children. In boys, iGCTs are more commonly located in the pineal region, whereas those reported in girls tend to involve the sellar region (6). To date, only six female cases of PPP associated with β-hCG-secreting iGCTs have been reported (**Table 3**) (9–14). One plausible explanation for the marked sex difference in PPP occurrence is the differential gonadal response to β-hCG. Tumor-derived β-hCG exerts potent LH-like activity but weak FSH-like activity because of its structural homology with LH. In boys, β-hCG activates the luteinizing hormone/choriogonadotropin receptor (LHCGR) on testicular Leydig cells, stimulating testosterone production and thereby inducing PPP (15, 16). However, the activation of ovarian function in girls generally requires the combined action of LH and FSH. Owing to the low FSH activity of β-hCG, triggering ovarian function is difficult; therefore, female children with iGCTs rarely present with PPP (6). iGCTs that cause PPP in girls mostly arise in the ovary. A literature review revealed the following possible reasons for the occurrence of PPP in female children with iGCTs: 1. β-HCG has weak FSH-like activity. Albert et al. proposed that a PB β-hCG concentration of 1000 mIU/mL is approximately equivalent to an FSH concentration of 2 IU/L (16). Extremely high β-hCG levels can also induce precocious puberty in females through combined LH- and FSH-like effects. 2. β-HCG can induce androgen production via the ovaries. β-HCG and LH share LHCGR on ovarian theca cells. At supraphysiological concentrations, they persistently activate the cAMP–PKA signaling pathway and upregulate the expression of steroidogenic enzymes, including CYP17A1, thereby promoting the synthesis of androstenedione and testosterone. When androgen levels become sufficiently elevated, these are directly converted to estrogens by aromatase from the adrenal glands (17, 18). 3. Some tumor cells exhibit aromatase activity, converting androgens to estrogens (14). Shibata et al. observed strong immunohistochemical staining for both hCG and aromatase in an intracranial choriocarcinoma from a girl with PPP, suggesting that the tumor cells themselves may have contributed to estrogen production through aromatase activity (13). 4. Hyperprolactinemia may arise through several mechanisms. Previous studies have suggested that hCG stimulates pituitary lactotroph activity, whereas β-hCG may also increase PRL indirectly by promoting estrogen production, because estrogen stimulates prolactin gene transcription and subsequent prolactin secretion. In patients with hypothalamic–pituitary stalk involvement, tumor infiltration or compression may impair hypothalamic dopamine delivery to the anterior pituitary, thereby relieving the tonic dopaminergic inhibition of lactotrophs and increasing PRL secretion (19, 20). Elevated PRL may promote pubertal development by (1) increasing ovarian responsiveness to FSH and LH; (2) increasing ovarian estrogen receptor expression and thereby augmenting estrogen activity; and (3) directly increasing ovarian responsiveness to hCG. 5. β-HCG-producing tumor cells can also produce E2, thereby leading to PPP (21).

**Table 3:** Six cases of β-hCG-secreting iGCTs among females presenting with PPP.

Reference	Age	Clinical manifestations	Tumor markers	Tumor location	Pathological type
Kubo O et al., 1977, Japan	5 years old	General convulsions, headache, nausea, vomiting, bilateral breast development	PB β-hCG 75 ng/mL, CSF β-hCG 146 ng/mL, E2: ND	Sellar region	Ectopic pinealoma
Kitanaka et al., 1994, Japan	6 years and 3 months old	Polyuria, polydipsia, headache, vomiting, decreased visual acuity, bilateral breast development	PB β-hCG 1800.5 mIU/mL, E2: 113.8–172.5 pmol/L	Sellar region	Immature teratoma
O’Marcaigh AS, 1995, United States	8 years old	Headache, vomiting, bilateral breast development, hirsutism	PB β-hCG 187 mIU/mL, CSF β-hCG 81 mIU/mL, E2: 228pmol/L	Sellar region	Germinoma
Starzyk J, 2001, Poland	5 years and 9 months old	Bilateral breast development, body odor, vaginal discharge and menarche	PB β-hCG 924.5 mIU/mL, AFP 20.8 ng/mL, E2: 783.4 pmol/L	Sellar region	-
Shibata N, 2021, Japan	7 years and 6 months old	Vomiting, anorexia, loss of consciousness, visual field disturbance, bilateral breast development	PB β-hCG 48800 mIU/mL, CSF β-hCG 28200 mIU/mL, E2:110.1 pmol/L	Sellar region	Choriocarcinoma
Chen H, 2022, China	4 years old	PPP	PB hCG 426.1 IU/L, E2: 116 pmol/L	Sellar region	-

NR, not reported; ND, not detectable.

On the basis of the aforementioned factors, PPP observed in the 3 girls in this study is considered to have a multifactorial etiology. β-HCG levels in the 3 girls in the present study and the 6 previously reported girls were very high. After treatment, β-hCG levels returned to normal, which was accompanied by regression of bilateral breast development and regression of the Tanner stage to Stage I, suggesting that precocious puberty was related mainly to excessively high β-hCG levels. Moreover, adrenal androgen levels were also elevated in cases 1 and 3. A portion of the excess adrenal androgens and/or testosterone can be converted into estrogens by aromatase. Furthermore, increased expression of aromatase by tumor cells may promote the conversion of androgens to estrogens. Both cases 2 and 3 had hyperprolactinemia, and their PRL levels decreased after treatment, which suggested that elevated PRL levels constitutes one of the underlying mechanisms contributing to PPP. In summary, multiple factors may jointly contribute to the occurrence of PPP in girls with iGCTs. In addition, Samson et al. reported that choriocarcinoma cells could produce E2 independently of testosterone (21). Many existing studies have reported that males with hCG-secreting tumors have gynecomastia (22–25), confirming that certain iGCTs can produce E2. In case 2, the serum E2 level was below the reporting threshold for the assay (<36.7 pmol/L) and therefore not quantifiable. However, the markedly low FSH and LH levels indirectly suggested feedback suppression by elevated endogenous estrogen levels. As no biopsy was performed, whether the tumor cells themselves secrete E2 or possess aromatase activity remains unclear. Among the 6 previously reported cases, 4 underwent pathological biopsy, yielding diagnoses of 1 immature teratoma, 1 choriocarcinoma, 1 atypical teratoma, and 1 germinoma. Future accumulation of additional pathological data may help clarify the histopathological subtypes and clinical characteristics of iGCTs.

Notably, after the full course of chemoradiotherapy was completed according to guidelines (8), tumor control in the 3 girls was satisfactory, β-hCG levels returned to normal, breast development regressed, and sex hormone levels decreased. The gonadotropin-like bioactivity of tumor-derived β-hCG may stimulate ovarian estrogen production, thus suppressing the HPGA through negative feedback. Following tumor treatment, sex hormone levels decrease. Sudden loss of this negative feedback inhibition may increase gonadotropin levels, which could subsequently trigger CPP (26). In this study, case 1 developed recurrent bilateral breast development 7 months after completing radiotherapy and was diagnosed with secondary CPP. On the basis of previous recommendations regarding the long-term management of PPP and the clinical findings of the present case series, we recommend long-term endocrine surveillance after the conclusion of antitumor treatment to detect secondary CPP and other endocrinopathies. Patients should attend follow-up appointments at least semiannually, with each visit including assessments of growth parameters, secondary sexual features and clinical symptoms, as well as serial endocrine biochemistry and bone age evaluation (3, 26).

The strength of this study lies in the consecutive characterization of 3 girls from a single center and a comparison with six previously reported cases, providing a systematic clinical overview of this extremely rare presentation. The principal contribution of the study is clinical rather than mechanistic, as it highlights a recognizable diagnostic pattern characterized by PPP, preceding polyuria and polydipsia, hypothalamic–pituitary involvement, elevated β-hCG levels, and additional pituitary dysfunction. Recognition of this pattern may prompt earlier evaluation for iGCTs in girls who present with precocious puberty and thereby reduce delayed diagnosis or misdiagnosis. Nevertheless, the small sample size and retrospective single-center design limit the generalizability of the findings. The lack of histopathological confirmation also precluded definitive tumor classification and direct assessment of tumor-derived estrogen secretion or intratumoral aromatase activity. Furthermore, ovarian steroidogenesis, prolactin-mediated ovarian effects, and other proposed mechanisms of precocious puberty were not directly evaluated. Therefore, the mechanistic explanations discussed in this study should be regarded as hypotheses derived from previous literature rather than as mechanisms established in our patients. Finally, water deprivation and desmopressin response tests were not performed, and primary polydipsia and partial arginine vasopressin resistance could therefore not be completely excluded. These limitations warrant cautious interpretation of the findings.

In conclusion, we report the clinical characteristics of 3 female children with β-hCG-secreting iGCTs who presented with PPP and AVP-D. In children presenting with precocious puberty accompanied by multiple pituitary dysfunctions, including AVP-D, or mass effect manifestations, such as decreased visual acuity, routine testing of serum β-hCG and AFP and contrast-enhanced sellar MRI are recommended to exclude organic lesions, including iGCTs. Furthermore, serial monitoring of endocrine axis hormones is needed to facilitate prompt symptomatic intervention in pediatric patients with iGCTs.

## Data Availability

The original contributions presented in the study are included in the article/supplementary material. Further inquiries can be directed to the corresponding author.
